# Transhiatal chest drainage in mediastinoscope and laparoscope-assisted esophagectomy for esophageal cancer: a retrospective study

**DOI:** 10.1186/s13019-022-01953-0

**Published:** 2022-08-24

**Authors:** Katsuji Hisakura, Koichi Ogawa, Yoshimasa Akashi, Jaejeong Kim, Shoko Moue, Yusuke Ohara, Yohei Owada, Shinji Hashimoto, Tsuyoshi Enomoto, Tatsuya Oda

**Affiliations:** 1 Department of Gastrointestinal and Hepato-Biliary-Pancreatic Surgery, Faculty of Medicine, University of Tsukuba, Tsukuba, Ibaraki 305-8575 Japan; 2 Department of Surgery, Hitachi, Ltd., Hitachinaka General Hospital, Hitachinaka, Ibaraki Japan

**Keywords:** Esophageal cancer, Pleural effusion, Transhiatal chest drainage, Mediastinoscope and laparoscope-assisted esophagectomy

## Abstract

**Background:**

Mediastinoscope and laparoscope-assisted esophagectomy for esophageal cancer occasionally causes postoperative accumulation of pleural effusion despite the preservation of the mediastinal pleura. Transhiatal chest drainage has been reported to be useful for thoracic esophagectomy; however, its use in mediastinoscope and laparoscope-assisted esophagectomy remains unelucidated. This study aimed to evaluate the effectiveness and safety of transhiatal chest drainage in mediastinoscope and laparoscope-assisted esophagectomy.

**Methods:**

This retrospective study included patients who underwent mediastinoscope and laparoscope-assisted esophagectomy for esophageal cancer from 2018 to 2021. Transhiatal chest drainage involved the insertion of a 19-Fr Blake® drain from the abdomen to the left thoracic cavity through the hiatus. We assessed its effectiveness and safety by the daily drainage output, accumulation of postoperative pleural effusion, frequency of postoperative thoracentesis, and other complications. The drainage group comprising 24 patients was compared with the non-drainage group comprising 13 patients, in whom a transhiatal chest drainage tube was not placed during mediastinoscope and laparoscope-assisted esophagectomy.

**Results:**

The median daily output of the transhiatal chest drainage was 230 mL on day 1, 385 mL on day 2, and 313 mL on day 3. The number of patients with postoperative pleural effusion was significantly reduced from 10/13 (76.9%) in the non-drainage group to 4/24 (16.7%) in the drainage group (*p* = 0.001). The frequency of thoracentesis in the drainage group was significantly lower than that in the non-drainage group (*p* = 0.002). There were no significant differences in the occurrence of other postoperative complications.

**Conclusions:**

Transhiatal chest drainage could evacuate pleural effusion effectively and safely after mediastinoscope and laparoscope-assisted esophagectomy.

## Background

Chest drainage is essential for full lung expansion in the postoperative management of esophagectomy due to the clinical consequences of pleural effusion [1]. Mediastinoscope and laparoscope-assisted radical esophagectomy for esophageal cancer is not performed through the thoracic approach [2, 3]. Theoretically, this surgical procedure limits and prevents the exposure of the pleural cavity. However, mediastinoscope and laparoscope-assisted esophagectomy also causes accumulation of pleural effusion despite the preservation of the mediastinal pleura, particularly in the left thoracic cavity [4]. Therefore, the placement of an effective chest drainage tube is essential for esophagectomy, even when a mediastinoscope and laparoscope-assisted approach is used.

Although the placement of an intercostal chest drainage tube is a standard practice in transthoracic esophagectomy, the intercostal drainage is inappropriate for the mediastinoscope and laparoscope-assisted approach, where the surgical field is far from the thoracic wall. Transhiatal chest drainage, reportedly used for transthoracic esophagectomy, can be inserted into the thoracic cavity via laparoscopy without manipulating the thoracic wall [5, 6]. However, no studies have assessed its use in mediastinoscope and laparoscope-assisted esophagectomy. Therefore, this study evaluated the effectiveness and safety of transhiatal chest drainage in mediastinoscope and laparoscope-assisted esophagectomy by comparing the drainage output, accumulation of postoperative pleural effusion, and the incidence of thoracentesis and other complications in patients with or without a transhiatal chest drainage tube.

## Methods

### Patient characteristics

This retrospective study included all patients who underwent mediastinoscope and laparoscope-assisted esophagectomy for esophageal cancer at the Tsukuba University Hospital from 2018 to 2021. We excluded patients who had a history of lung surgery, converted to thoracotomy, developed air leakage from the lung, and required tube drainage at the time of wound closure, underwent simultaneous surgery for a second cancer, or whose gastric conduit could not be used.

### Procedure

**Fig. 1:**
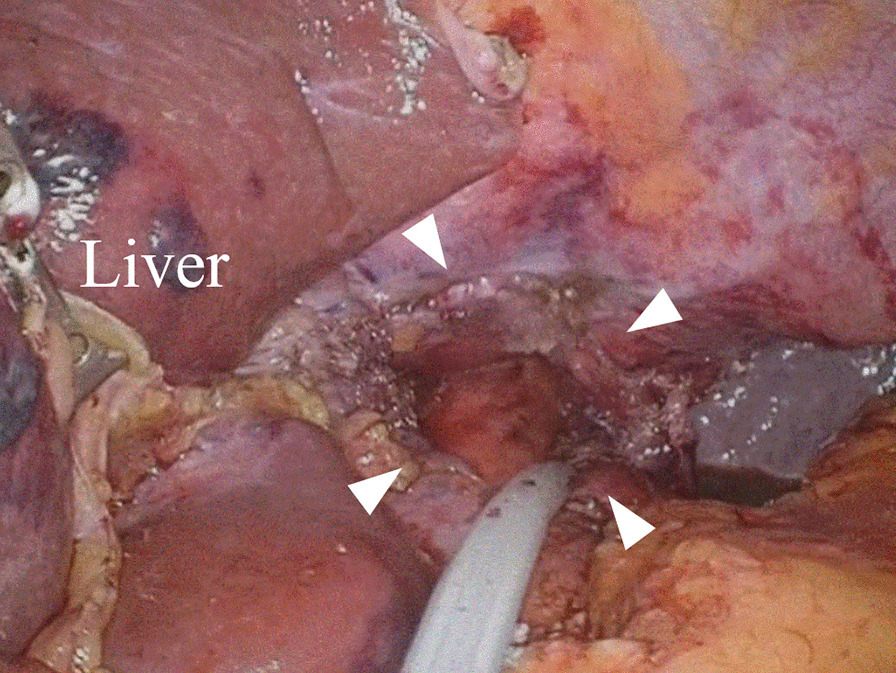
Intraoperative photograph showing the Blake drain inserted from the inferior hepatic space to the left thoracic cavity through the hiatus. Arrowhead, esophageal hiatus

**Fig. 2:**
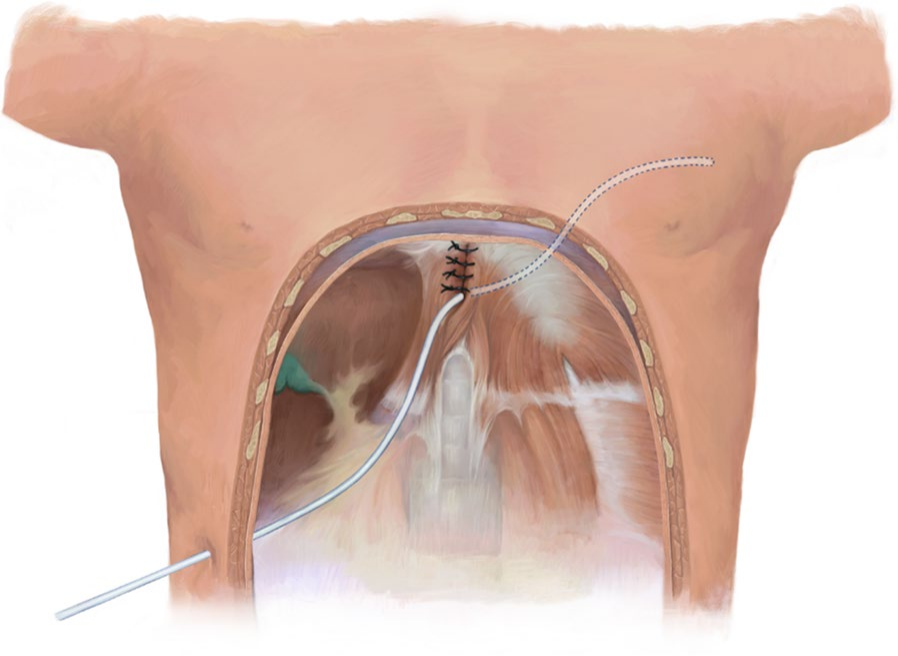
Representation of the intraoperative insertion of the Blake drain from the inferior hepatic space to the left thoracic cavity through the hiatus. Arrowhead, esophageal hiatus

**Fig. 3:**
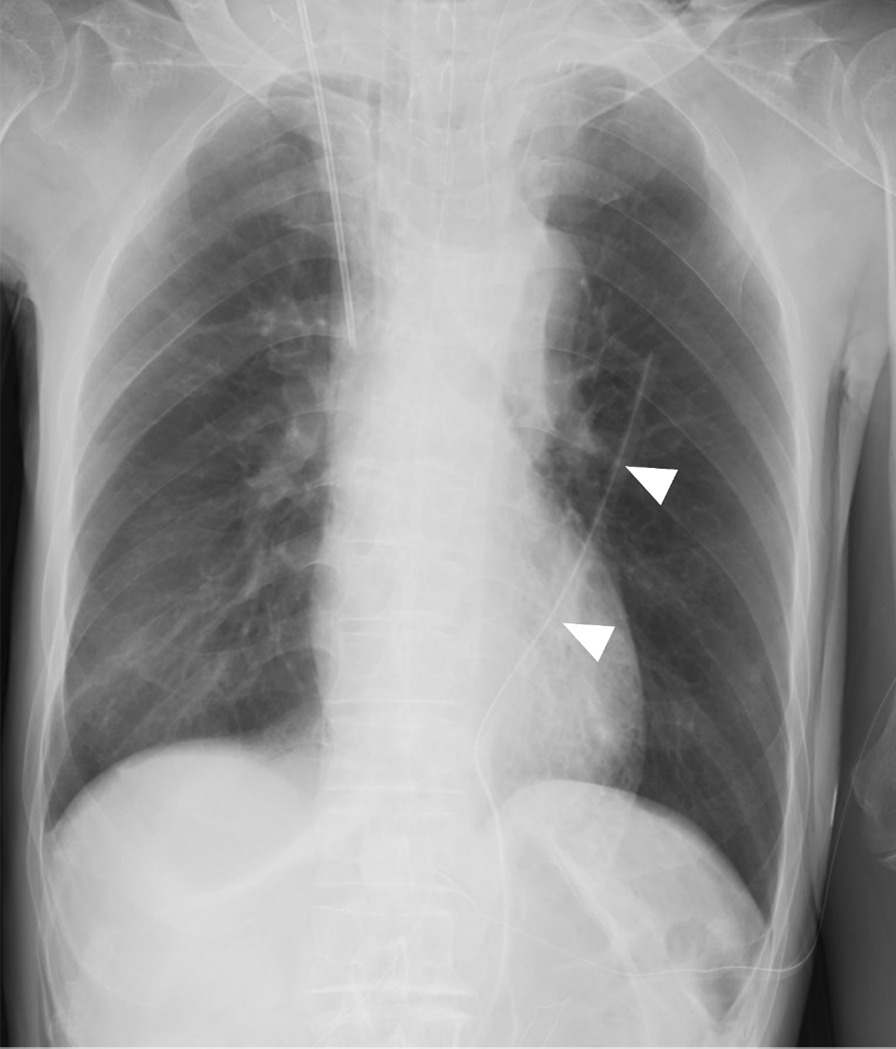
The postoperative chest radiography. Arrowhead, transhiatal left pleural drainage tube

All patients underwent mediastinoscope-assisted transcervical esophagectomy with radical lymph node dissection, followed by the laparoscopic transhiatal approach by a single surgeon, without the thoracic approach as described previously [2, 3]. In almost all patients, abdominal and transhiatal procedures were performed laparoscopically, while the procedures were hand-assisted in seven patients. In this study, patients were divided into two groups (with or without transhiatal chest drainage). In the drainage group, the transhiatal left chest drainage tube was placed after esophagectomy and lymph node dissection. The drainage tube was inserted from the inferior hepatic space to the left thoracic cavity through the hiatus by intentional left pleural incision before hiatal closure (Figs. 1, 2). In the non-drainage group, the pleura was preserved, and the hiatus was closed directly. We used a 19-Fr Blake® (Ethicon, Inc., Somerville, NJ, USA) drain connected to a portable vacuum system (J-Vac®; Ethicon). Drainage tube was placed only in the left side because a previous study demonstrated that pleural effusion after this surgery frequently accumulated at the left side by the anatomical location of the esophagus [4]. Reconstruction was performed using a gastric conduit via the retrosternal route with cervical anastomosis, considering the possibility of locoregional recurrence. A review of the video database revealed that transhiatal chest drainage, introduced in February 2020, has been used in all patients since. Patients operated from 2018 to January 2020 were assigned to the non-drainage group, and patients operated from February 2020 to 2021 were assigned to drainage group (Fig. 3).

To assess the effectiveness and safety of transhiatal chest drainage, the primary outcome measure was daily drainage output and accumulation of postoperative pleural effusion. The secondary outcome was the frequency of postoperative thoracentesis and other complications. Chest radiography was performed everyday until postoperative day 5 to evaluate the accumulation of pleural effusion. The indication for postoperative thoracentesis was the accumulation of pleural effusion, which was diagnosed by radiography and required oxygen administration. Thoracentesis was avoided for patients who could not be punctured safely on ultrasonography. Other data, including age, sex, body mass index (BMI), tumor location, and T stage based on the 7th edition of the AJCC/UICC TNM classification system [7], lymph node metastasis status, histological type, neoadjuvant chemotherapy, operation time, blood loss volume, number of dissected lymph node, frequency of postoperative thoracentesis and its complications, drained volume of pleural effusion, occurrence of postoperative complications (grade 3 or more) based on the Clavien–Dindo classification [8], duration of oxygen administration, percentage of body weight loss on postoperative day (POD) 14, and duration of postoperative hospital stay, were also collected from the clinical database. Decision of thoracentesis was made by the conference between the intensive care unit staff and surgeons based on the patient’s respiratory status and X-ray findings. In the drainage group, the procedure time to insert the transhiatal drainage tube was measured. The study’s retrospective protocol was approved by our institutional review board (No. R1-017), and written informed consent for publication was obtained from the patients.

### Statistical analysis

Comparisons between the drainage and non-drainage groups were performed using the Chi-squared test for categorical variables (sex, tumor location, T stage, lymph node metastasis, histological type, and postoperative complications) and the Mann–Whitney U test for continuous variables (age, BMI, operation duration, blood loss volume, number of dissected lymph nodes, duration of oxygen administration, and postoperative hospital stay). All analyses were performed using IBM SPSS Statistics for Windows version 25.0 (IBM Corp., Armonk, NY, USA). *P*-values < 0.05 were considered statistically significant.

## Results

### Patient demographics

During the study period, 44 patients underwent mediastinoscope and laparoscope-assisted esophagectomy. We excluded 7 patients owing to their history of lung surgery (n = 1), conversion to thoracotomy (n = 2), air leakage from the lung at wound closure (n = 2), simultaneous surgery for hypopharyngeal cancer (n = 1), and an unusable gastric conduit (n = 1). A total of 37 patients were enrolled. A transhiatal drainage tube was placed intraoperatively in 24 patients (drainage group), and in the other 13 patients, a drainage tube was not placed (non-drainage group). The clinical characteristics of the patients were shown in Table 1. There were no significant differences in clinical parameters between two groups.

**Table 1:** Clinical data of the drainage and non-drainage groups

	Drainage n = 24	Non-drainage n = 13	*p*-value
Age, years (IQR)^a^	70 (69–74)	71 (67–75)	0.96
Male/female	20/4	8/5	0.14
BMI^b^ (IQR)	23.0 (20.0–25.1)	22.0 (18.9–23.4)	0.26
Thoracic esophagus/esophagogastric junction	14/10	9/4	0.51
cT 1/2/3/4	6/7/9/2	2/5/6/0	0.20
cN (+)/(−)	16/8	9/4	0.97
Squamous cell carcinoma/adenocarcinoma	12/12	9/4	0.26
Neoadjuvant chemotherapy	18 (75.0%)	11 (84.6%)	0.50

^a^Interquartile range

^b^Body mass index

### Intraoperative outcomes

Table 2 showed intraoperative outcomes. The median and interquartile range (IQR) time to insert the transhiatal drainage tube was 4 (3–5) minutes in the drainage group. All 24 patients enrolled in the drainage group were able to undergo transhiatal chest drainage tube insertion as confirmed by postoperative radiography. There were no significant differences in operative time, blood loss, and the number of resected lymph nodes between the groups. All patients underwent R0 resection.

**Table 2:** Operative data of the drainage and non-drainage groups

	Drainage n = 24	Non-drainage n = 13	*p*-value
Drain insertion time (IQR)^a^	4 (3–5)	–	–
Operation time, min (IQR)	425 (348–480)	415 (390–459)	0.97
Blood loss volume, mL (IQR)	110 (52–191)	124 (79–158)	0.83
Dissected nodes (IQR)	39 (33–54)	38 (28–47)	0.63

^a^Interquartile range

### Postoperative outcomes

**Fig. 4:**
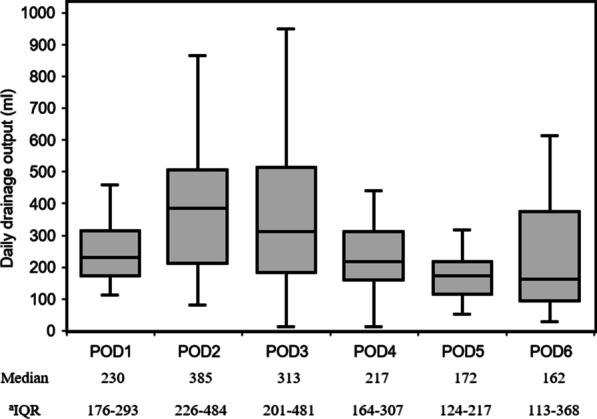
Daily drainage output of pleural effusion. ^a^Interquartile range

The daily drainage output was shown in Fig. 4. The median volume in the drainage group was 230 mL on day, 1, 385 mL on day 2, and 313 mL on day 3. The number of patients drained over 350 mL/day was 19/24 (79.2%). The median volume of drainage output decreased to < 200 mL after postoperative day (POD) 5. The transhiatal drainage tube was removed after a median time of 6 PODs. The postoperative data were shown in Table 3. In the non-drainage group, the accumulation of pleural effusion was observed on chest radiography in 10/13 (76.9%). In contrast, the number of patients significantly decreased to only 4/24 (16.7%) in the drainage group. Thoracentesis was required 7 times for 6 patients in the non-drainage group until POD 2. The median (IQR) volume evacuated by one thoracentesis was 450 (375–600) mL in the non-drainage group. One patient in the non-drainage group developed pneumothorax after thoracentesis. All 6 patients required left-side thoracentesis. In contrast, one patient in the drainage group, who also had anastomotic leakage, required right thoracentesis. This number was significantly less than that in the non-drainage group (*p* = 0.002). Further, the patient in the drainage group did not require left thoracentesis, which also varied from the findings in the non-drainage group (*p* = 0.001). There were no significant differences between both groups in postoperative complications, duration of oxygen administration, and length of postoperative hospital stay.

**Table 3:** Postoperative data of the drainage and non-drainage groups

	Drainage n = 24	Non-drainage n = 13	*p*-value
Accumulation of pleural effusion	4 (16.7%)	10 (76.9%)	0.001
Thoracentesis	1 (4.2%)	6 (46.2%)	0.002
Left	0	6	0.001
Right	1	0	0.47
Median volume of thoracentesis, mL (IQR)^a^	450	450 (375–600)	
Pneumothorax caused by thoracentesis	0 (0%)	1 (7.7%)	0.17
Other complications	8 (33.3%)	4 (30.8%)	0.87
Respiratory	3 (12.5%)	1 (7.7%)	0.65
Anastomotic leakage	3 (12.5%)	1 (7.7%)	0.65
SSI^b^	3 (12.5%)	1 (7.7%)	0.65
Chylothorax	0 (0%)	1 (7.7%)	0.17
Duration of oxygen administration, days (IQR)	3 (1–4)	4 (2–6)	0.40
Postoperative hospital stay, days (IQR)	18 (13–21)	14 (12–18)	0.30

^a^Interquartile range

^b^Surgical site infection

## Discussion

Transhiatal chest drainage in mediastinoscope and laparoscope-assisted esophagectomy could be performed intraoperatively in a short time to evacuate postoperative pleural effusion effectively and reduce the frequency of atelectasis requiring postoperative thoracentesis, which carries the risk of iatrogenic pneumothorax. Furthermore, the transhiatal chest drainage system did not employ an underwater seal but employed a portable vacuum system, which was reportedly easy to insert and safe in terms of other complications. Despite being unable to control the drain tip position from the transabdominal view, pleural effusion was evacuated effectively. Currently, no study has discussed the suitability of an indwelling transhiatal chest drainage tube in mediastinoscope and laparoscope-assisted esophagectomy. To our best knowledge, this study is the first to assess the effective and safe evacuation of pleural effusion via intraoperative placement of a chest drainage tube in mediastinoscope and laparoscope-assisted esophagectomy.

A single Blake drain inserted through the abdominal wall effectively evacuated pleural effusion in this study. Moreover, the placement of the transhiatal chest drainage tube was a simple procedure, which was completed in < 6 min. The utility of a mediastinal drainage tube from the abdominal wall through the hiatus after thoracic esophagectomy has been reported [9]. Transhiatal chest drainage using a Blake drain has also been reported to be effective and safe in Ivor Lewis esophagectomy [6]. The insertion of a chest drainage tube intraoperatively via the intercostal space is obviously difficult in mediastinoscope and laparoscope-assisted esophagectomy, and in such cases, transhiatal chest drainage is appropriate.

The median volume of drainage output by transhiatal chest drainage was approximately 300 mL for 3 days postoperatively. Daily physiological pleural fluid filtration is estimated to be 350 mL/day [10, 11]. Since the number of patients drainaged over 350 mL/day was 19 (79.2%), transhiatal chest drainage would have been effective for these patients. Furthermore, approximately the same amount of pleural effusion was drained by thoracentesis in more than half of the patients in the non-drainage group. Therefore, in these patients, transhiatal chest drainage may have been effective. In contrast, the adequate duration of drainage postoperatively was unclear in this study. In the non-drainage group, thoracentesis was required until POD 2. In the drainage group, the median volume of drainage output decreased to < 200 mL after POD 5. The indwelling period for the drainage tube could be potentially shortened.

In this study, we used a 19-Fr Blake drain connected to a portable vacuum system. The use of a portable vacuum system connected to the Blake drain is a viable alternative to an underwater seal [6, 12, 13]. A single Blake drain inserted from the right intercostal space into the left thoracic cavity across the mediastinum is useful for the drainage of the left pleural effusion after thoracic esophagectomy [14]. Transhiatal chest drainage in mediastinoscope and laparoscope-assisted esophagectomy had similar effects to these drainage systems. Despite the description of the use of a 15-Fr drain in other studies [6, 14], there was little leakage around the drainage tube when using a 19-Fr drain, suggesting little tube obstruction. The 15-Fr drain is more beneficial than the 19-Fr one in terms of preventing dislocation, excessive drainage, and pain caused to patients [14]. Further clinical experiences are needed to clarify the appropriate drain size.

This study had some limitations. First, this study was retrospective with a small sample size. Therefore, patient characteristics, including sex, histological types, and progression, were not homogenous. Second, this study did not compare transhiatal chest drainage with conventional thoracic drainage. However, the inferiority of thoracic drainage might be definitive owing to the difficulty of the thoracic approach in mediastinoscope and laparoscope-assisted esophagectomy. Third, transhiatal chest drainage could not improve the clinical outcomes associated with oxygenation, including the duration of oxygen administration, respiratory complications, and postoperative hospital stay, although the patients were spared from thoracentesis. Further studies are required to validate these findings.

## Conclusions

We demonstrated that transhiatal chest drainage was an effective, safe, and simple procedure to evacuate pleural effusion after mediastinoscope and laparoscope-assisted esophagectomy. Moreover, this procedure may reduce the frequency of postoperative thoracentesis. A large, prospective randomized controlled trial is required to completely assess the benefits and limitations of transhiatal chest drainage.

## Data Availability

Not applicable.

## Abbreviations

BMI: Body mass index
IQR: Interquartile range
POD: Postoperative day
SSI: Surgical site infection
